# The Active *plus *protocol: systematic development of two theory- and evidence-based tailored physical activity interventions for the over-fifties

**DOI:** 10.1186/1471-2458-8-399

**Published:** 2008-12-04

**Authors:** Maartje M van Stralen, Gerjo Kok, Hein de Vries, Aart N Mudde, Catherine Bolman, Lilian Lechner

**Affiliations:** 1 Open University of the Netherlands, Department of Psychology, PO Box 2960, 6401 DL, Heerlen, the Netherlands; 2 School for Public Health and Primary Care (Caphri), Maastricht University, PO Box 616, 6200 MD Maastricht, the Netherlands; 3 Maastricht University, Department of Psychology, PO Box 616, 6200MD Maastricht, the Netherlands; 4 Maastricht University, Department of Health Promotion, PO Box 616, 6200 MD, Maastricht, the Netherlands

## Abstract

**Background:**

Limited data are available on the development, implementation and evaluation processes of physical activity promotion programmes among older adults. More integrative insights into interventions describing the planned systematic development, implementation and evaluation are needed.

**Methods and design:**

The purpose of this study is to give an integrative insight into the development of the Active *plus *programme applying the six-step Intervention Mapping protocol. The Active *plus *programme consisted of two theory- and evidence-based tailored physical activity promotion interventions, both comprising three tailored letters delivered over four months and aimed at raising awareness of insufficient physical activity, and stimulating physical activity initiation and maintenance among the over-fifties.

The first intervention, the basic tailored intervention, provided tailored letters that intervened on the psychosocial determinants of physical activity. The second intervention, the intervention *plus*, provided the same tailored information but additionally provided tailored information about physical activity opportunities in the specific environment in which the older adults lived. This environment-based component also provided access to a forum and e-buddy system on a website. A plan for implementation and evaluation is also described.

**Discussion:**

The planned development of the Active *plus *programme resulted in two theory- and evidence-based tailored physical activity interventions targeted at the over-fifties.

**Trial Registration:**

Dutch Trial Register NTR 920

## Background

Regular physical activity (PA) reduces the risk of health problems such as cardiovascular diseases, obesity, and type 2 diabetes [1-7]. Moreover, these disorders become more prevalent and their impact increases when people age [1]. Currently in the Netherlands, only 58.6% of the general population meets the international health guideline [8,9] that recommends that people are moderately physically active at least five days per week for a minimum of 30 minutes per day [4]. The amount of older adults complying with this guideline is even lower [8,9], declining to less than 50% among adults aged 75 and over [9]. Together with the fact that older adults form a large percentage of the population – in the Netherlands 32% of all residents are over 50 years of age [8], a percentage that is expected to rise even further in the coming years [8,10] – stimulating PA among this large and growing group is of major relevance.

Several authors have reviewed the research literature with respect to interventions aimed at promoting PA among older adults [11-14], and concluded that, despite difficulties in comparing the studies, the promotion of PA among older adults is feasible. However, while the majority of the evaluation studies report on the effectiveness of the programme, they provide insufficient information about the theory- and evidence-based programme development; such as a description of the determinants, the linked theoretical methods and how the theoretical methods are put into practice via practical intervention strategies [15,16]. Consequently, adequate programme evaluation is limited [17] and identification of effective working mechanisms to promote PA in older adults is hardly possible. A systematic approach to the development of an intervention leads to more effective interventions [18,19]. Therefore, this paper aims to contribute to the need for integrative descriptions of interventions by describing the systematic planned theory- and evidence-based development of the Active *plus *programme, including two computer-tailored PA interventions. This paper does not provide the results of the evaluation of the interventions, but it will focus on their systematic development. For this purpose, the Intervention Mapping (IM) protocol was applied [20].

## Method and design

IM is a six-step protocol that facilitates a stepwise process for theory- and evidence-based development of health promotion interventions [20]. The six steps include: (1) a needs assessment of the study population and the definition of programme objectives; (2) defining the performance objectives, specifying what changes are needed; (3) selecting theory-based intervention methods and practical strategies to change health behaviour and its determinants; (4) developing an intervention programme in which all strategies are integrated, as well as selecting, testing and producing intervention materials; (5) developing a programme adoption and implementation plan; and (6) anticipating a process and effect evaluation of the programme. In this paper we describe our approach to each step of the IM protocol applied to the promotion of PA among older adults, focusing particularly on steps 1–4.

### Step 1: Needs assessment

The first step in IM concerns a needs assessment [20], which was made for PA among older adults via a combination of methods, such as a search of the literature, focus-group interviews with the target group (adults aged 50 and over) and additional interviews with intermediates, such as project leaders at the Municipal Health Organizations and public servants from the health promotion departments of local authorities. A search of the literature led to the identification of lack of physical activity as a risk behaviour and older adults as the target population in preventing chronic illnesses [1-7]. Furthermore, regular PA is particularly important for older adults to enable them to maintain their mobility [21] and independence [22], improve muscle strength [23] and mental and emotional wellbeing [24], and prevent falls [25]. Six focus-group interviews with a total of 47 adults aged 50 and over were conducted according to predefined protocols [26]. These interviews gained further in-depth knowledge of the needs of older adults with regard to PA, PA stimulation and PA interventions in addition to the findings of the literature. From the needs assessment three intended programme outcomes emerged. The programme outcomes were based on the PA guideline and the PA recommendations set by the American College of Sports Medicine and the American Heart Association, promoting 30–60 minutes moderate-intensity aerobic activity per day in older adults [4]. Specifically, older adults who do not reach the international PA guideline of at least 30 minutes per day on at least five days of the week should become aware of their insufficient PA level, and increase and maintain a new PA level. Second, older adults who do reach the PA guideline, but are physically active for less than 60 minutes per day, should increase their PA level to at least 60 minutes per day, and maintain this new PA level. Third, older adults who are PA for more than 60 minutes per day at a moderate intensity should maintain this sufficient level of PA.

### Step 2: Matrices of change objectives

In the second step, expected changes and performance objectives as a result of the intervention were specified and important and changeable personal and external determinants were selected. The first task was to translate the previously mentioned risk behaviour, namely a lack of physical activity, into health-promoting behaviour. Based on a literature search and focus-group interviews with the target group and intermediates, two ways of increasing PA were identified. First, recreational PA was encouraged, such as playing sports, walking and cycling. Second, increasing the amount of PA in people's daily routines was promoted, for instance by stimulating walking and cycling for transport and stimulating PA at work (e.g. by taking the stairs instead of the lift, going for a walk during lunch breaks or walking over to speak to colleagues instead of using e-mail) and at home (e.g. while doing gardening, chores and household activities).

The second task of step 2 was to specify the two behaviours into performance objectives, which are the exact behavioural outcomes of the programme expected from the target group [20]. Specification of the performance objectives was done via a combination of methods, namely a review of the literature [27], a Delphi study among 118 international experts in the field of health promotion and/or in the field of PA determinants [28], and a review of theoretical models, such as the I-Change model [29-31], the Health Action Process Approach [29-34], the self-regulation theory [35-37] and the self-determination theory [38,39]. Eight performance objectives were specified for each programme objective. As an illustration, the performance objectives for the first PA behaviour – namely recreational PA – are presented in Table 1.

**Table 1:** Performance objectives for awareness raising, initiation and maintenance of recreational PA among older adults

1. older adults monitor their recreational PA level
2. older adults indicate reasons to be physically active as recreation
3. older adults identify solutions to take away the barriers to being physically active for recreation
4. older adults decide to become more recreationally physically active
5. older adults make specific plans to become more recreationally physically active
6. older adults increase their recreational PA
7. older adults make specific plans to cope with difficult situations occurring while being recreationally physically active
8. older adults maintain their recreational PA by enhancing their routine and preventing relapses

As a third element of step 2, a structured analysis was conducted to assess the most relevant and changeable personal and environmental determinants associated with awareness-raising and the initiation and maintenance of PA behaviour [20]. The methods used were the above-mentioned review of the scientific literature on the determinants of initiation and maintenance of PA [27], and a Delphi study on determinants of awareness, initiation and maintenance of PA among international experts in the field of PA determinants and/or in the field of health promotion theories [28]. Furthermore, in focus-groups interviews with older adults the determinants of awareness-raising and PA initiation and maintenance, as well as intervention strategies to stimulate these determinants were discussed. The determinants of awareness-raising, initiation of PA and maintenance of PA were identified and categorized as personal and (social and physical) environmental determinants. Furthermore, two groups of new theoretical developments regarding determinants related to behavioural change were identified, in order to further improve PA interventions. First, recent research had provided evidence that post-motivational psychological constructs, like action plans, coping plans and relapse prevention and recovery skills, are effective in translating intentions into actions [40-46]. Including intervention strategies that stimulate these post-motivational constructs might be important in changing PA behaviour. Second, researchers acknowledged the importance of a more ecological approach that also addresses social and physical environmental determinants in influencing PA in addition to psychological motivational determinants. Humpel and colleagues found that improved perceptions of the PA opportunities in the neighbourhood were associated with increased walking levels [47]. Sallis et al (2007) also found that the PA behaviour of adults aged 50 or over was more affected by perceived environmental determinants than by the PA behaviour of younger adults [48]. Consequently, interventions aimed at developing realistic perceptions of what is feasible with respect to PA in their environment might be helpful in changing the PA behaviour of the over-fifties. Taking the aforementioned aspects into account, a decision was taken to develop two interventions, both addressing post-motivational determinants, with one intervention (the intervention *plus*) additionally addressing several environmental determinants. These determinants are shown in the first columns of Additional File 1 and 2.

Finally, a matrix was developed that linked the performance objectives and their hypothesized determinants, resulting in specific learning and change objectives. Selected examples of the matrix of change objectives are shown in Table 2.

**Table 2:** Examples of the matrix of performance objectives related to changes in behavioral and environmental determinants with regard to recreational PA

**Performance objectives**	**Determinants**			
	
	**Awareness**	**Knowledge**	**Attitude**	**Self-efficacy**
**1. Older adults monitor their recreational PA level**	Older adults become aware of their own recreational PA level	Older adults know the PA recommendations and learn how to compare their own PA level with the recommendations		
	Older adults monitor and report their own recreational PA			

**2. Older adults indicate reasons to be physically active as recreation**	Older adults become aware of their personally relevant benefits of being sufficiently physically active	Older adults learn about the health benefits of sufficient PA and can name personally relevant reasons for being sufficiently physically active	Older adults feel positive about being sufficiently physically active	

**3. Older adults identify solutions to take away the barriers to being physically active for recreation**	Older adults become aware of the situations and barriers that prevent them from being sufficiently physically active	Older adults learn how to identify difficult situations and learn about solutions that can take away the barriers		Older adults feel confident about being able to take away and to cope with their barriers

### Step 3: Theory-based methods and practical strategies

In the third step of IM, theoretical methods and practical strategies that had previously been found or were likely to change the identified determinants were selected [20]. A theoretical method is a general technique or process that is derived from theories and that can be applied to influence behavioural determinants [20]. For example, modelling is a theoretical method derived from the Social Cognitive Theory [49] that can be applied to influence the older adults' self-efficacy and perceptions of social influence [20]. A practical strategy is a specific application of a theoretical method tailored to the target population and the intervention setting [20]. For example, in the Active *plus *programme, a practical strategy used for the application of modelling was to include at least two role model stories per letter, including pictures of active similar others (of the same age and gender) together with a quote about their motivation for being active, situations which they found hindered being active and how they coped with them, or their plans to become more active or stay active. When translating the theoretical methods into practical strategies, it is important to meet the theoretical conditions that apply to the method used [50]. For example, Social Cognitive Theory states that modelling is only effective when the participants meet certain conditions, such as identification with the model; reinforcement of the model; and the demonstration of adequate skills by the model to the participant.

Theoretical methods, practical strategies and their conditions were derived from a search of the literature [20], existing intervention programmes like the Vitalum intervention [51] and focus-group interviews with the target group. Theoretical methods used in the two tailored Active *plus *interventions were: personalization, tailoring, feedback, argumentation, persuasive communication, self-monitoring, consciousness-raising, active learning, social modelling, and shifting perspectives. The determinants, their linked theoretical methods and practical strategies and the tools used in both the intervention programmes for increasing daily PA routine levels are shown in Additional file 1. The determinants, theoretical methods and practical strategies additionally used in the intervention including community information, the intervention *plus *group, are presented in Additional file 2.

### Step 4: Producing programme components and materials

Based on the methods and practical strategies, a programme plan was developed, including the scope and sequence of the Active *plus *programme and a list of the required materials [20]. First, several brainstorming sessions with experts and members of the target group were conducted to identify the intervention materials that had to be developed to match the objectives and practical strategies. Since the older section of the target group was less comfortable with the use of computers and the internet, it was decided that the data would be collected via written questionnaires and the tailored messages would be sent as printed letters.

Second, a computer-tailored intervention was considered to be the most appropriate framework, since it would facilitate the delivery of intervention programmes to a large number of older adults and the tailoring of the programme materials to the individual characteristics and interests of the participants. With regard to health promotion programme development, several studies have shown the promising effects of the computer-tailoring method [52-55]. Computer-tailoring is a health education technique that adapts health education strategies and information to a specific person, by addressing characteristics specific to this individual derived from an individual assessment through a computerized process [53,56]. Computer-tailored interventions were found to be more effective in changing health behaviour than non-tailored interventions [55]. This can be explained by the findings that more personal tailored messages are more likely to be read, understood, remembered and discussed with others, and are rated higher, saved more often and evaluated as more interesting than non-tailored messages [56-59]. The development of computer-tailored interventions requires: (1) a data source, including the significant characteristics of the person derived from an individual assessment such as a questionnaire; (2) a message library that contains the intervention messages that may be needed; (3) a set of decision rules that selects messages matched to the needs of a specific person; and (4) a channel, such as a letter, that delivers the messages to the specific person [53]. The programme materials and intervention messages were developed, combined and included in two tailored intervention programmes. This resulted in two computer-tailored PA interventions targeted at the over-fifties, which included multiple computer-tailored feedback letters, addressing several intervention strategies.

**Figure 1:**
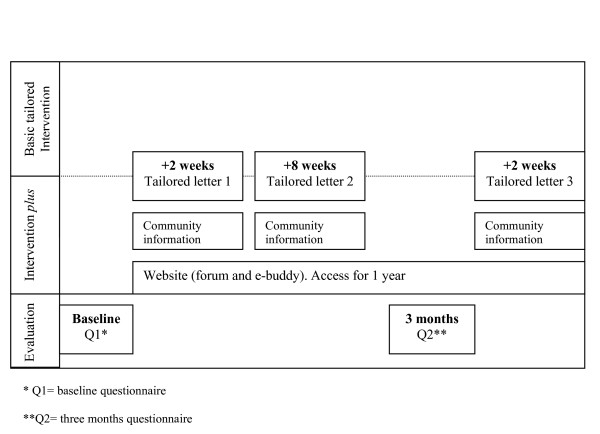
**Overview of the intervention programmes**. * Q1 = baseline questionnaire. **Q2 = three months questionnaire

Figure 1 shows the timing of the distribution of the questionnaires and tailored letters. The first and second tailored letters were based on the personal data gathered at baseline. The third letter was based on the data gathered at baseline and after three months, adressing the changes the specific older adult had undertaken in these three months. There were two intervention groups. The first intervention group, the basic tailored intervention condition, received tailored letters that intervened based on the psychosocial determinants of PA. The second intervention group, the intervention *plus *group, got the same tailored information but additionally received tailored information about PA opportunities in their specific environment combined with access to a forum and e-buddy system on a website.

#### Tailored letter 1 and 2

The first and second tailored letter were based on the personal data gathered at baseline and were sent within, respectively, two and eight weeks of the first questionnaire. The messages sent depended on the stage of change of an individual at baseline. Individuals could be divided into five older adults groups, namely: (1) precontemplators – older adults who did not reach the PA guideline and who did not want to initiate PA; (2) contemplators – older adults who did not reach the PA guideline and who wanted to initiate PA within six months; (3) preparators – older adults who did not reach the PA guideline and who wanted to initiate PA within one month; (4) actors and maintainers (<60 min.) – older adults who did reach the PA guideline but were physically active for less than 60 minutes per day; and (5) actors and maintainers (≥ 60 min.) – older adults who did reach the PA guideline and who were active for more than 60 minutes per day.

**Figure 2:**
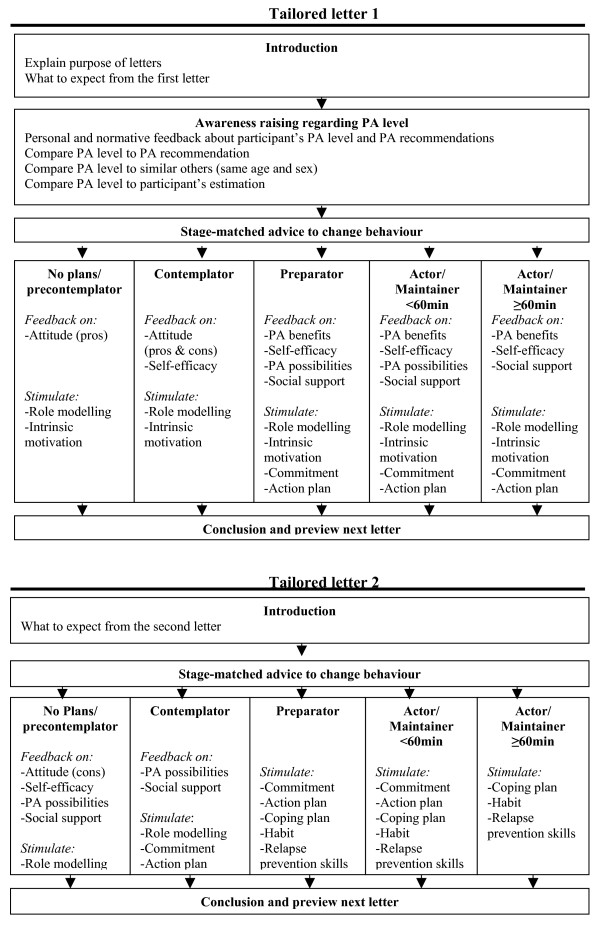
Tailored print communication elements of first and second tailored letter.

The content of the first and second letter per stage group is shown in Figure 2. Precontemplators received feedback in the first letter on their PA behaviour, to raise awareness of their own physical inactivity. They received feedback on their intention to become physically active and on their perceived pros, in which the pros they perceived were confirmed and other not perceived pros were listed. Further, they received role model quotes about intrinsic reasons for becoming physically active, after which they were stimulated to write down their own reasons in order to increase their intrinsic motivation. In the second letter, they received feedback about the perceived cons of being physically active, in which perceived cons were weakened. They received feedback and role model stories about their self-efficacy to become more physically active, after which solutions to difficult situations were presented. Further, feedback about their perceived social support was given, and seeking social support and a sports partner was encouraged. In addition, they received practical information about which activities in their daily routine, or recreational activities/sports, they could adopt.

Contemplators received feedback in the first letter on their PA behaviour to raise awareness of their own physical inactivity, followed by feedback on their intention to become more physically active and on their perceived pros and cons. Participants received role model stories and feedback on their perceived self-efficacy to become more physically active. Further, after receiving role model stories, the participants were stimulated to write down what might intrinsically motivate them to become more physically active. In the second letter, feedback about perceived social support, followed by encouragement to seek social support and a sports partner, was given. Additionally, participants received practical information about PA possibilities they could incorporate into their daily routine or take up as recreational activities/sports, and were encouraged to formulate one or more action plans to stimulate the translation of their intentions into action and to encourage commitment.

The first letter to the preparators consisted of feedback on their own physical inactivity in order to raise awareness, on the participant's intention to become more physically active, and on the benefits of increasing one's PA level, as well feedback and role model stories on their perceived self-efficacy levels. Feedback about their perceived social support was provided, after which seeking social support and a sports partner was promoted. Role model stories were given, followed by encouragement to define their intrinsic motivation for becoming physically active. Further, they received practical information about which activities to include in their daily routine or take up as recreational activities/sports, followed by encouragements to formulate one or more action plans in order to stimulate PA initiation and commitment. In the second letter they were prompted to formulate action plans again and to make coping plans in order to deal with difficult situations they encountered. By stimulating the new PA behaviour to become an automatic, repeated and goal-oriented behaviour, the formation of PA as a habit was encouraged [60-62]. Further, participants were trained to enhance their relapse prevention skills in order to prevent and to cope with relapses.

In their first letter, actors and maintainers (<60 min) received compliments for having a sufficient PA level and feedback to raise their awareness of their PA and their intention to become more physically active. They also got feedback about the benefits of further increasing their PA level to 60 minutes per day. In addition, role model stories and feedback about their perceived self-efficacy to stay physically active, as well as encouragement to define their intrinsic motivation, were provided. Solutions to difficult situations were presented in order to enhance further PA initiation. Feedback and encouragement to seek social support was provided. Furthermore, actors and maintainers received practical information about activities they could adopt followed by encouragement to formulate one or more action plans in order enhance further their initiation of and commitment to PA, and to stimulate habit formation. In the second letter the formulation of action plans to further increase habit formation, and coping plans in order to deal with difficult situations they had encountered, were stimulated. Further, participants were trained to increase their relapse prevention skills.

In their first letters, actors and maintainers (≥ 60 min) received compliments for having a sufficient PA level and feedback to raise their awareness of their PA level and their PA intentions. They also got role model stories and feedback about their perceived self-efficacy to maintain their PA level and perceived social support. Solutions to difficult situations were presented in order to prevent lapses. Further, role model stories and encouragement to write down their personal motivation to stay physically active were provided. Additionally, they were prompted to formulate one or more action plans to stimulate habit formation and to increase commitment. In the second letter they were stimulated to formulate coping plans in order to deal with difficult situations they might encounter. Habit formation was further promoted, and training given in relapse prevention skills in order to prevent and cope with lapses.

Participants in the intervention *plus *condition (the intervention including community information), in the first letter additionally received further information about a website and its e-buddy system and forum, handouts describing walking and cycling routes in their own neighbourhoods, examples of exercises to do at home, and contact information for sports clubs in their neighbourhoods that matched their interests and abilities. In addition to the second letter, participants again got information about the website and its e-buddy system and forum, and handouts describing new walking and cycling routes in their neighbourhoods. Examples of exercises to do at home and contact information for sports clubs matched to their interests were also sent to the participants if this met their needs and if they had not received this information in the first letter (precontemplators and contemplators).

#### Tailored letter 3

**Figure 3:**
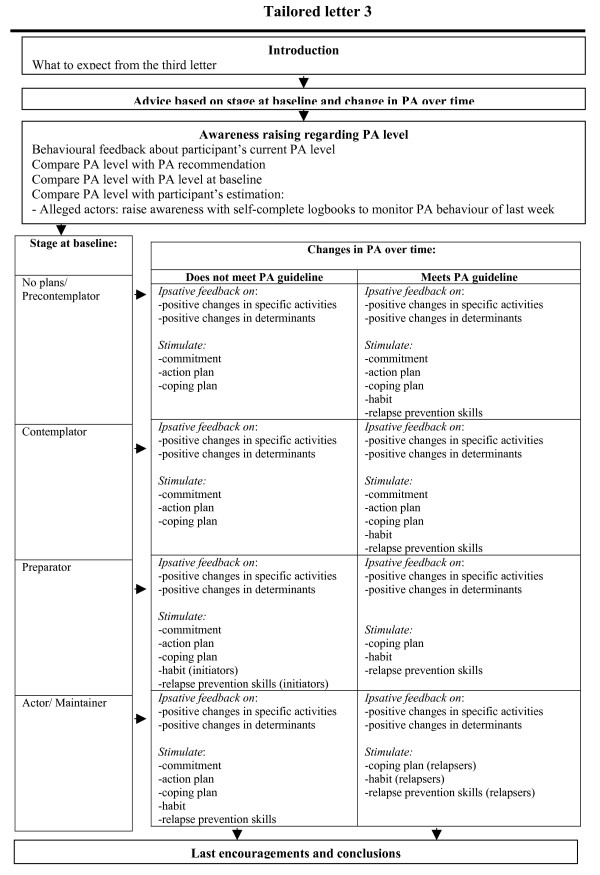
Tailored print communication elements of third tailored letter.

The third tailored letter was based on a combination of the personal data gathered at baseline and the results of the second questionnaire three months later. The contents of the third letter for each stage group are shown in Figure 3. The messages depended on the stage of the individual at baseline and on changes in the PA level and determinants of the participant over the three months between the first and second questionnaire. Improvements were rewarded and possible relapses were addressed appropriately with additional suggestions to again increase PA levels.

All participants in the intervention *plus *condition, the intervention including community information, were additionally given information about the website and its e-buddy system and forum. They also got handouts describing new walking and cycling routes in their neighbourhoods, and maps of their close neighbourhoods on which walking and cycling possibilities were highlighted.

#### Pre-testing of materials

The three letters sent by the two intervention programmes, the intervention strategies used and the additional community information materials were pre-tested on appreciation and use in a pilot study of 57 older adults [63]. The letters, intervention strategies and community information materials were subsequently adjusted according to the preferences of the pilot group. This resulted in two three-staged tailored intervention programmes ready to be implemented and tested for effects in a larger study design.

### Step 5: Adoption and implementation

In the fifth step, a plan for the implementation of the two intervention programmes was developed. A linkage system within each community district was formed that connected the developers of the intervention with representatives of the future users of the intervention, for example a project leader from the regional Municipal Health Organization, a public servant for health promotion at the local authority, and a member of the senior citizens' league [20]. This linkage board was responsible for the adoption and implementation of the intervention programmes and for the recruitment of the participants. Based on the pilot study, it was concluded that the best way to recruit participants was to invite them to participate via a personalized letter by mail. The Active *plus *program was approved by the Medical Ethics Committee of Maastricht University and the University Hospital Maastricht, and is registered with the Dutch Trial Register (NTR920).

### Step 6: Evaluation planning

In the sixth step of the IM protocol, an evaluation plan and the data collection measurements were developed [20]. The evaluation included a process and effect evaluation. The evaluation was designed following the CONSORT statement, which is a checklist and flow diagram for reporting randomized controlled trials. The effect evaluation was performed to determine whether the intervention programmes were successful in changing the PA behaviour of the participants when compared to the waiting list control group, which was subjected to the interventions at the end of the programme at 12 months. In addition, it was performed in order to assess whether the intervention *plus *programme – including community information – was as effective in changing PA as the less costly intervention, the basic tailored intervention without community information. Further, it was performed to identify which working mechanisms were effective in promoting PA in older adults. The effects were measured using a pre-post test randomized control trial design, including two intervention groups and one waiting list control group, with a three-month (during the intervention), six-month (short-term) and 12-month (long-term) follow-up. The main outcome measure was level of PA behaviour. Process evaluation was carried out to collect data on the use, appreciation and subjective impact of the tailored letters, practical strategies and community information among the participants of the intervention groups.

## Discussion

For the development of the Active *plus *programme, consisting of two tailored PA interventions targeted at older adults, the IM protocol was used. A systematic approach to development, implementation and evaluation is needed in order to increase the likelihood of an effective intervention [18,19] and to optimally draw conclusions about effective or ineffective interventions and intervention strategies. Although IM is a time-consuming process, it provides a useful protocol for the stepwise development of interventions and the planning of the implementation and evaluation of intervention programmes. We found that the use of IM helped to utilize theory and empirical evidence into an intervention. It was perceived as a useful checklist which, when systematically followed, helps to minimize mistakes and ensures that all important objectives are covered. Also, since new concepts were integrated into the interventions, such as self-regulation determinants and community information, the IM protocol was a practical and relevant aid.

In conclusion, the systematic development of the Active *plus *programme resulted in two theory- and evidence based tailored interventions aimed at raising awareness and stimulating initiation and maintenance of PA among Dutch older adults.

This paper aimed to demonstrate how tailored PA promotion programmes can be developed in a systematic way, including a community-based intervention, according to the IM protocol. Further, the paper aimed to provide an insight into the programme, performance, learning and change objectives, intervention strategies used, programme materials, participant recruitment, implementation and evaluation of the two tailored interventions. The results of the effectiveness of the study, which will be reported in other papers, may contribute to the knowledge about the effectiveness of PA stimulation amongst older adults, the influence of awareness of the environment on changes in PA, the possibilities of computer tailoring, and effective intervention strategies for enhancing PA among the over-fifties. In addition, if the developed intervention programmes are found to be effective in changing PA, we have two interventions at our disposal that are ready to be implemented on a larger scale.

## Abbreviations

PA: physical activity; IM: Intervention Mapping protocol.

## Competing interests

The authors declare that they have no competing interests.

## Authors' contributions

MVS, HDV, AM, CB and LL developed the concept and design of the study. MVS designed the interventions and coordinated the implementation of the interventions, data collection and data analyses of the study. HDV, AM, CB and LL provided support during the development and execution. MVS, GK and LL significantly contributed to writing this manuscript, while HDV, AM and CB were involved in revising the manuscript. All authors have read and approved the final version of the manuscript.

## Pre-publication history

The pre-publication history for this paper can be accessed here:



## Supplementary Material

Additional file 1 **Determinants and the theoretical methods, practical strategies and tools used in both interventions to increase recreational PA.** Click here for file

Additional file 2 **Determinants and the theoretical methods, practical strategies and tools used only in the intervention *plus *to increase recreational PA.** Click here for file
