# Supplementary material for: The Active plus protocol: systematic development of two theory- and evidence-based tailored physical activity interventions for the over-fifties
Source: BMC Public Health. 2008 Dec 4;8:399. doi: 10.1186/1471-2458-8-399 (PMC2613403; doi:10.1186/1471-2458-8-399)
Supplement: Additional file 1 — Determinants and the theoretical methods, practical strategies and tools used in both interventions to increase recreational PA. [file 1471-2458-8-399-S1.doc]

**Additional File 1. Determinants and the** theoretical methods, practical strategies and tools used in both interventions to increase recreational PA

| **Personal determinant** | **Theoretical method** | **Practical strategy** | **Tools** |
| --- | --- | --- | --- |
| Awareness | Tailored feedback | Provide personal and normative feedback | Computer-tailored advice in text and graphic format with feedback about participant’s PA level |
|  | Consciousness raising | Compare PA level with similar others, PA recommendation and current PA level | Computer-tailored advice in text and graphic format in which participant’s PA level is compared with similar others (same age and sex), the PA recommendation and participant’s estimation |
|  | Self-monitoring | Encourage monitoring of own behaviour | Self-complete logbooks to monitor own PA behaviour in last week |
| Knowledge | Tailored feedback and information delivery | Provide tailored feedback about PA recommendations, PA benefits and PA possibilities | Computer- tailored feedback in text about PA recommendations, health benefits of sufficient PA and PA possibilities (recreational, daily PA). |
| Commitment | Active learning | Encourage formulation of action plans | Post-its with spaces to write down plans to be PA and advice to commit to these plans. |
| Attitude | Feedback and argumentation | Provide personal feedback and arguments about pros and cons. | Computer-tailored feedback in text on perceived positive and negative consequences of PA. New arguments to change opinions are provided in text. |
|  | Reinforcement | Provide ipsative feedback on changes in attitude: evaluation of changes | Computer-tailored feedback in text on positive changes in attitude towards PA at follow-up |
| Self-efficacy | Feedback and argumentation | Provide personal feedback and new arguments on self-efficacy. | Computer-tailored feedback in text on difficult situations. New arguments to cope with these situations are provided. |
|  | Reinforcement | Provide ipsative feedback on changes in self-efficacy: evaluation of changes | Computer-tailored feedback in text on positive changes in perceptions of difficult situations at follow-up. |
|  | Social modelling | Provide role model stories about difficult situations and how to cope. | Picture of similar others (same age and sex) with quotes about a similar perceived difficult situation and how the role model coped |
| Intention | Feedback | Provide personal feedback on intention | Computer-tailored feedback in text on the participant’s intention to be physically active |
|  | Reinforcement | Provide ipsative feedback on intention: evaluation of changes | Computer-tailored feedback in text on positive changes in intention to be physically active |
| Intrinsic Motivation | Active learning | Invite to formulate motivation | Post-its with space to write down (intrinsically motivated) reasons to be physically active |
|  | Social modelling | Provide role model stories about intrinsic motives to be PA | Picture of a similar other (same age and sex) with quotes about their (intrinsic) motive to be physically active |
| Action planning | Active learning | Invite to formulation action plan | Post-its with space to write down plan to be physically active (what, when, with whom, where to be physically active) |
|  | Social modelling | Provide role model stories about action planning | Picture of similar other (same age and sex) with story about how specific planning helped them to initiate/maintain PA |
| Coping planning | Active learning | Invite to formulate coping plans | Figure with space to formulate “if-then” rules. “If” this difficult situation occurs, “then” I cope by .... |
| Relapse prevention skills | Shifting perspectives/ active learning | Encouraged to formulate an implementation intention plan to train in relapse prevention skills | Figure with motivational text to prevent lapses and to train in skills for coping with a lapse |
| Habit | Persuasive communication | Provide information and arguments to enhance habit | Information and new arguments in text to stimulate PA to become a habit (automatic, repeated and goal-oriented behaviour) |
|  | Active learning | Invite to formulate action and coping plans to enhance habit | Post-its with space for formulation of action plans (to stimulate automatic, repeated and goal-oriented behaviour) and a figure for formulation of coping plans (to break automatic stimulus-response relations). |

| **External determinant** | **Theoretical method** | **Practical strategy** | **Tools** |
| --- | --- | --- | --- |
| Social influence | Social modelling | Provide role model stories about PA motives, difficult situations and reinforcement | At least two pictures of similar others (same age and sex) with quotes about their PA motivation, perceived difficult situations and how to cope, specific planning and/or how they succeeded in initiating/ maintaining PA |
|  | Feedback and persuasive communication | Provide feedback and arguments on social support | Computer-tailored feedback in text on perceived social support and having a sports partner. New arguments on seeking social support and inviting a sports partner are provided |
|  | Reinforcement | Provide ipsative feedback: evaluation of changes | Computer-tailored feedback in text on positive changes in social support at follow-up |
