# Supplementary material for: The Active plus protocol: systematic development of two theory- and evidence-based tailored physical activity interventions for the over-fifties
Source: BMC Public Health. 2008 Dec 4;8:399. doi: 10.1186/1471-2458-8-399 (PMC2613403; doi:10.1186/1471-2458-8-399)
Supplement: Additional file 2 — Determinants and the theoretical methods, practical strategies and tools used only in the intervention plus to increase recreational PA. [file 1471-2458-8-399-S2.doc]

**Additional file 2. Determinants and the theoretical methods, practical strategies and tools used only in the intervention *plus*** to increase recreational PA

| **Determinant** | **Theoretical method** | **Practical strategy** | **Tools** |
| --- | --- | --- | --- |
| Perceived social environment or having a sports partner | Linking members to new networks of people | Providing e-buddy system and e-forum | Website with access to a forum to get in touch with participants living in the same neighbourhood (e-forum) and the option to invite sports partners (e-buddies) by e-mail |
| Perceived physical environment | Facilitating (cues, reinforcement, resource availability, consciousness raising) | Providing contact information sports clubs | Computer-tailored advice about contact information for sports clubs in the immediate neighbourhood that matches people’s interest |
|  |  | Providing walking and cycling routes | Handout showing walking and cycling routes in the immediate neighbourhood |
|  |  | Providing visual representation of walking and cycling possibilities in neighbourhood | Map (taken from Google Earth©) of neighbourhood with PA possibilities highlighted |
|  |  | Providing exercises to do at home | Examples of strength exercises at home |
